# *In Situ* Atomic-Scale Imaging of Interfacial Water under 3D Nanoscale Confinement

**DOI:** 10.1021/acs.nanolett.1c01092

**Published:** 2021-05-13

**Authors:** Manuel R. Uhlig, Ricardo Garcia

**Affiliations:** Instituto de Ciencia de Materiales de Madrid, CSIC, c/Sor Juana Inés de la Cruz 3, 28049 Madrid, Spain

**Keywords:** Solid−liquid interfaces, interfacial water, capillary condensation, nanoscale water bridges, atomic force microscope

## Abstract

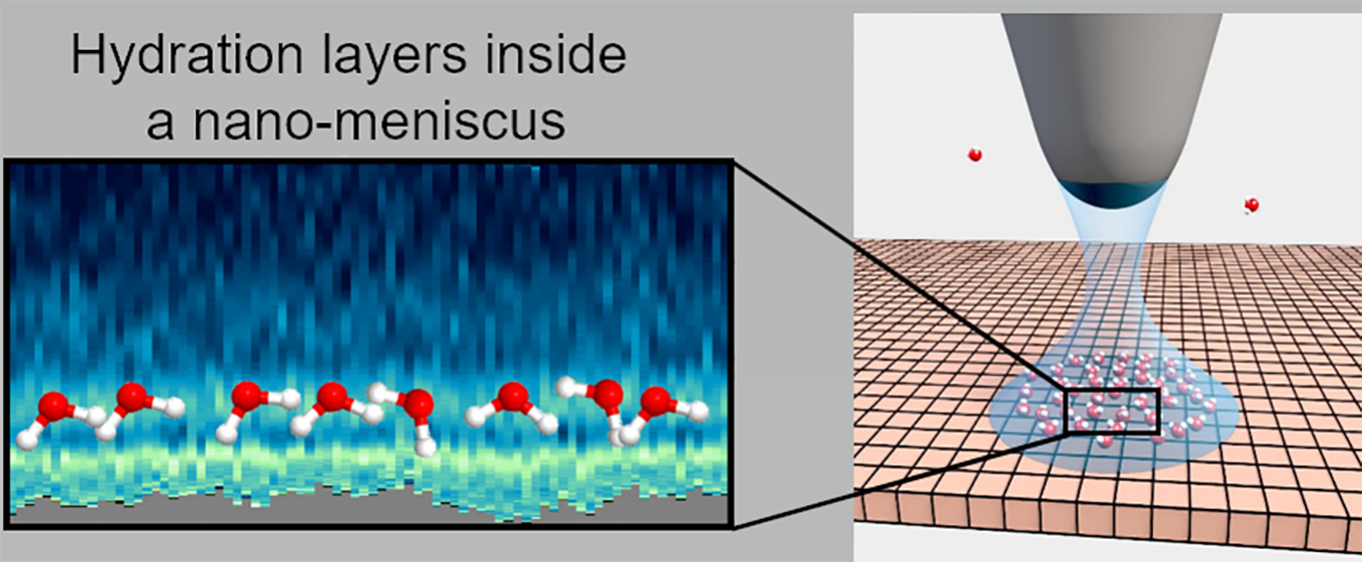

Capillary condensation of water from vapor is an everyday phenomenon which has a wide range of scientific and technological implications. Many aspects of capillary condensation are not well understood such as the structure of interfacial water, the existence of distinct properties of confined water, or the validity of the Kelvin equation at nanoscale. We note the absence of high-spatial resolution images inside a meniscus. Here, we develop an AFM-based method to provide *in situ* atomic-scale resolution maps of the solid–water interface of a nanomeniscus (80–250 nm^3^). The separation between the first two hydration layers on graphite is 0.30 nm, while on mica it is 0.28 nm. Those values are very close to the ones expected for the same surfaces immersed in bulk water. Thus, the hydration layer structure on a crystalline surface is independent of the water volume.

Capillary condensation of water from vapor is a common phenomenon that happens in the gap between two neighboring solid surfaces under some thermodynamic conditions. It has many implications in different fields such as tribology,^1^ adhesion,^2^ nanolithography,^3^ and fuel recovery.^4^ Despite its ubiquitous nature, some relevant features of capillary condensation are poorly understood. The structure of interfacial water,^5,6^ the very existence of distinct properties of confined water,^7,8^ the validity of the Kelvin equation at nanoscale,^9,10^ and the relationship between capillary adhesion and friction^11^ are aspects of capillary condensation under debate.

At ambient relative humidity (RH) values, i.e., those in the 30–70% range, the Kelvin equation predicts capillary condensation in sub-20 nm cracks, pores, or irregularities of a surface.^12,13^ The small size and three-dimensional geometry of those nanostructures has prevented high-spatial resolution imaging of the resulting solid–water interface. Different approaches were undertaken to circumvent the above problem. Initially, experiments were performed in liquid water confined between two large mica surfaces (surface force apparatus).^12,14,15^ Later, AFM setups were implemented to measure the adhesion force of nanoscale water bridges^1,16^ or to study solid–water interfaces.^17^ Recently, water trapped between a solid surface and a large 2D materials surface (graphene or hexagonal BN) was used to study the properties of confined water.^18−20^ In all the above experimental setups, the separation between the solid surfaces was either sub-5 or sub-1 nm. The use of lithographic methods provides some devices where the spatial confinement in the *xy* axes was in the 200 nm range while in *z* it might reach the sub-1 nm range.^20^ It can be said that the above-mentioned experimental setups do not provide direct high-spatial resolution (vertical and lateral) measurements of the solid–water meniscus interface.

We have developed an atomic force microscope (AFM) method to map the interfacial water structure inside a nanoscale water bridge (nanomeniscus). In the experimental setup, a sharp silicon tip and a flat surface, either hydrophobic (graphite) or hydrophilic (mica), are placed in a sealed chamber with humidity and temperature controls. The experimental process has three main steps, i.e., formation of a nanomeniscus, atomic-scale imaging of the interfacial water structure inside the nanomeniscus, and transformation of the AFM observables into force–distance maps of the interface.

Figure 1 shows some force–distance curves (FC) obtained on a graphite surface in humid air and immersed in ultrapure water. When both the tip and the graphite are immersed in water (Figure 1a), the force–distance shows an oscillatory behavior profile (Figure 1b). Specifically, the force shows the presence of oscillatory and monotonically decaying terms. The periodicity of the oscillation arises from entropic effects associated with the molecular packing of the liquid which implies local changes in the density of the liquid near the solid surface. The monotonic term comes from cohesive interactions between the liquid molecules and the interactions of the liquid with the solid surfaces.^17,21^ This phenomenon happens very close to the solid surface (e.g., within 1 nm from the surface). On the other hand, the formation of a nanoscale water bridge between the tip and the surface modifies the force–distance profile^22^ and increases the adhesion force.^1^ The force–distance curve of a nanomeniscus has three main regions (Figure 1c,d). Far from the surface the interaction force is negligible (region 1). By approaching the tip toward the surface, we reach the conditions for the spontaneous nucleation of a water meniscus (region 2). Eventually the tip enters into mechanical contact with the surface (region 3).

**Figure 1:**
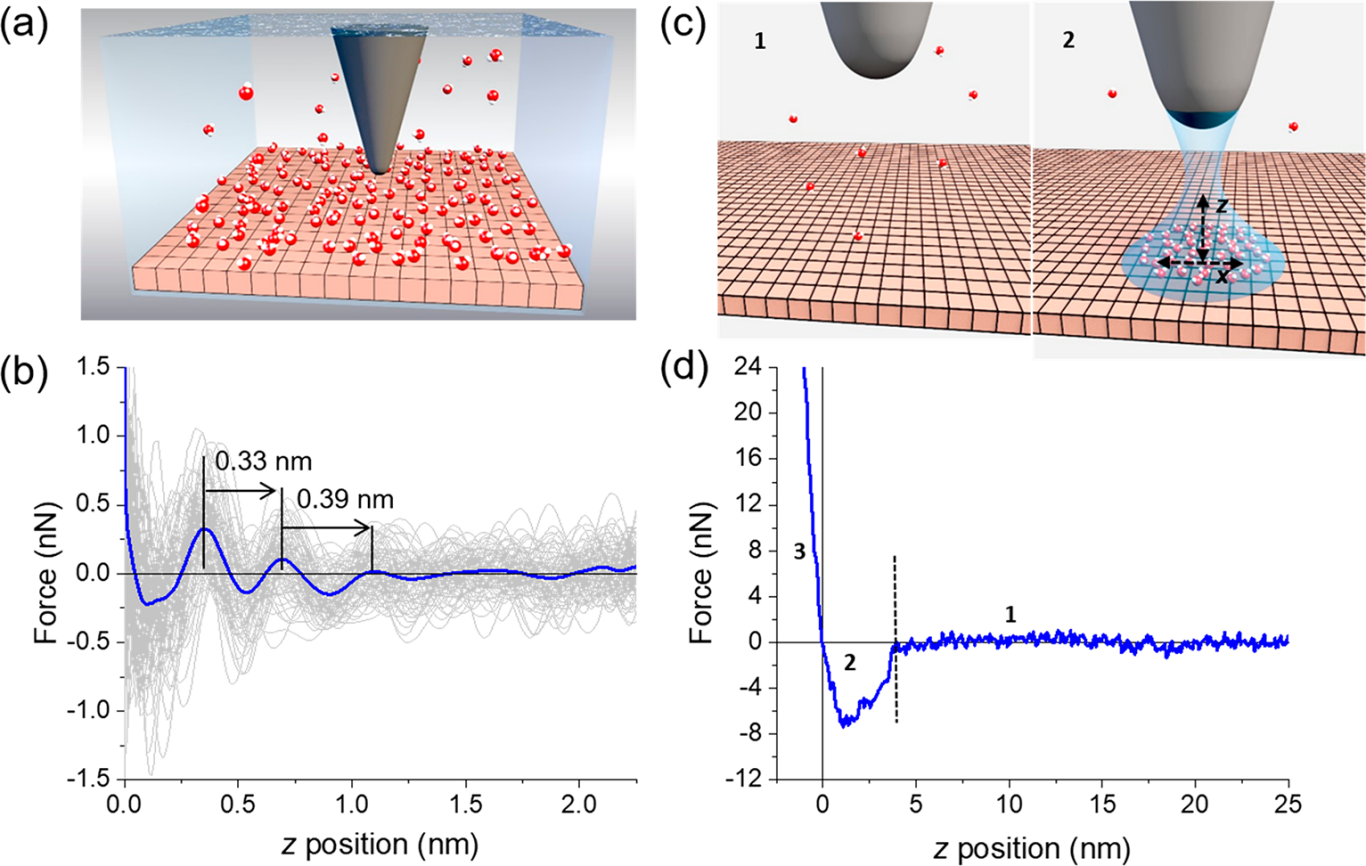
Force–distance curves in water and inside a nanomeniscus. (a) Scheme of an AFM experiment in bulk water. (b) Experimental force–distance curve measured on a pristine graphite surface immersed in bulk water. The monotonic component of the force is not shown. (c) Scheme of a tip–nanoscale water meniscus interface. The scheme shows the interface before (region 1, tip far from the surface) and after the formation of the nanomenicus (region 2, tip closer to the surface). (d) Experimental force–distance curve associated with the spontaneous formation of a nanoscale water meniscus between an AFM tip and a graphite surface. Experimental parameters: (b) *f*_2_ = 933 kHz, *k*_2_ = 1916 N/m, *Q*_2_ = 20.4, *A*_0_ = 103 pm, *A*_sp_ = 79 pm. (d) *k*_0_ = 42.2 N/m.

Molecular dynamics (MD) simulations show that menisci involving several thousands of water molecules are formed in 10 ps and might reach the equilibrium state in about 1 ns.^23,24^ Those times are several orders of magnitude shorter than our experimental *z*-step time (20 μs). For all purposes, the tip might be considered still while the water bridge nucleates.

The nucleation process is marked by a sudden drop in the value of the force, which gives rise to a capillary adhesion well (Figure 1d). The strength of the sudden drop in the force is controlled by the relationship between the cantilever force constant and the derivative of the capillary force. The width of the well determines the length of the nanomeniscus while the minimum in the force determines the adhesion force. The van der Waals interaction between the tip and the surface also contributes to the adhesion force, although the adhesion force is dominated by the capillary force component.^1^ Eventually, the tip enters into mechanical contact with the solid surface, which gives rise to a sharp repulsive region associated with the mechanical contact between tip and surface. To avoid the formation of large nanoscale water bridges, the experiments are performed at RH in the 30–75% range (*T* = 300 K).

The direct observation of hydration layers on hydrophobic materials such as graphene, graphite, and other graphite-like surfaces immersed in water (Figure 1b) is a very rare event.^25,26^Figure 1b might be considered a canonical FC of interfacial water on a pristine and uncontaminated graphite surface.^25^ However, more often than not the interlayer spacings measured on a graphite surface immersed in water are larger than those shown in Figure 1b.^25,27^ Graphite-like surfaces once cleaved and exposed to ambient air are contaminated by airborne hydrocarbon molecules.^25,28^

Figure 2a shows a 2D force map that includes two interfaces, an air–water bridge interface and a water bridge-graphite interface (Figure 1c). The map was generated by measuring the force from the AFM observables as a function of the tip–surface distance (Δ*z* = 15 nm) across the *x* coordinate (Δ*x* = 5 nm) (see Methods). The same pattern is repeated along the *x* coordinate. This observation underlines the reproducibility of the nanoscale water bridge formation. Figure 2b shows the FC distance (average value) extracted from Figure 2a. The curve is divided in three main regions, namely, air, inside the nanomeniscus, and graphite-hydration layers. The depth of the adhesion well makes it hard to identify the hydration layer structure near the graphite surface. A model of the nanomeniscus is shown in Figure 2c.

**Figure 2:**
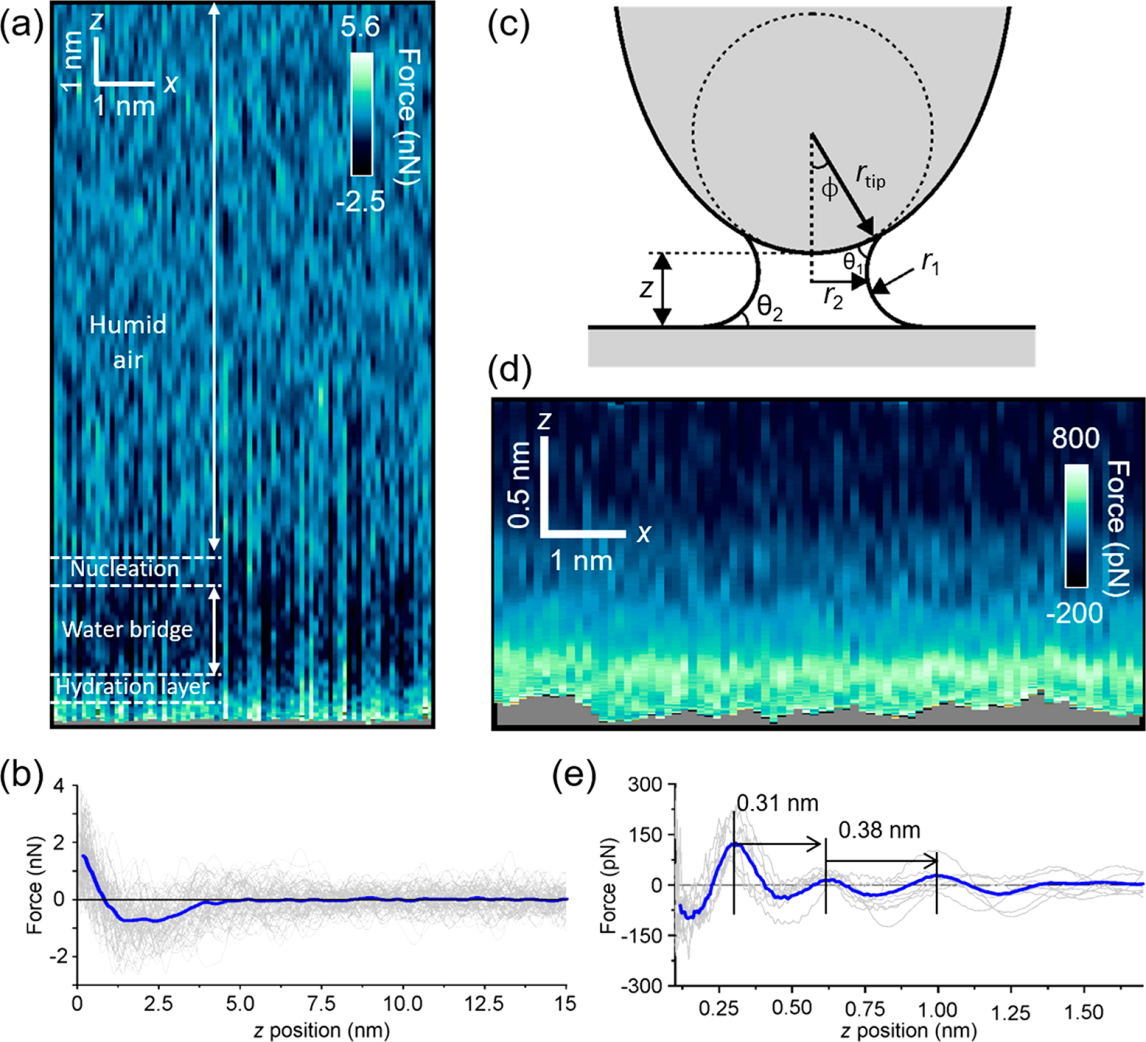
Two-dimensional force maps of nanoscale water bridges. (a) 2D force (*x, z*) map that includes both the tip–air–graphite and the tip–water bridge–graphite interface. (b) Force–distance curve extracted from Figure 2a. (c) Geometry of the nanomeniscus. (d) *In situ* 2D force map (*x, z*) of a graphite–nanoscale water bridge interface. (e) Force–distance curve extracted from Figure 2d. The FCs along the different *x* positions of the map are plotted in gray. The thick blue line is the average value. The monotonic component of the force is not shown. Additional experimental parameters: (a, b) *k*_0_ = 37.4 N/m, (d, e) *f*_2_ = 1.866 MHz, *k*_2_ = 2086 N/m, *Q*_2_ = 968, *A*_0_ = 627 pm, *A*_sp_ = 393 pm.

To obtain atomically resolved images of the solid–water interface inside the nanomeniscus, the tip is displaced in the *xz* plane without breaking the nanomeniscus. The 2D *xz* force map was obtained by confining the Δ*z* displacement within a 1.5 nm region from the solid surface (Figure 1c). Thus, the *z*-displacement is performed within the capillary adhesion well boundaries (see below). The map alternates molecular layers of high and lower force values.

Figure 1c shows a scheme of the AFM method to characterize the solid–nanoscale water bridge interface. Figure 2d shows a 2D force map obtained inside a nanomeniscus. Figure 2e shows the oscillatory component of the FC extracted from the force map (Figure 2d). The FC shows an oscillating behavior with distances similar to the ones measured when a pristine graphite surface is fully immersed in ultrapure water (Figure 1b).

To quantify the interfacial water structure inside a nanoscale water bridge, we have imaged several nanomenisci on graphite and mica surfaces (Figure 3). On a pristine graphite surface, the interface shows several layers (Figure 3a,b). The force–distance curves show an oscillatory behavior that alternates attractive and repulsive regions until the tip contacts with the graphite surface. The interlayer spacing between the first and second layer is 0.29 nm. Those values are very close with the ones predicted by MD simulations on pristine graphite surfaces.^25,29,30^ Furthermore, the oscillations observed in the perpendicular component of the force are consistent with a MD simulation result on the capillary adhesion of a meniscus formed between two parallel plates.^24^ The airborne contamination of the graphite surface might also be affected by the adsorption of water from the air.^22^

**Figure 3:**
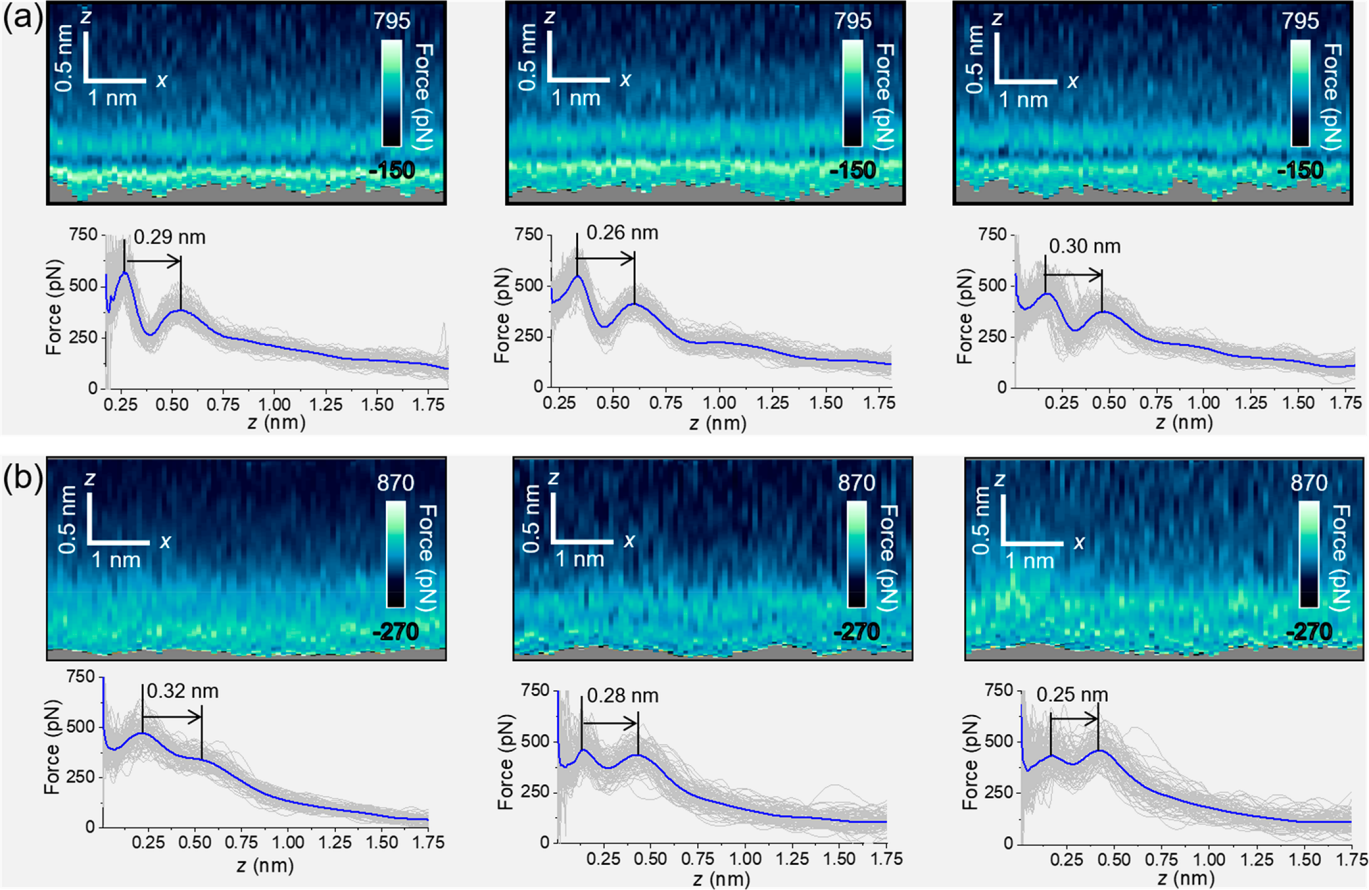
*In situ* 2D force maps and force–distance curves of graphite and mica–nanoscale water bridge interfaces. (a) 2D force (*x*, *z*) maps of different graphite–nanoscale water bridge interfaces. Each map is accompanied by its force–distance curve (average). (b) 2D force maps of different mica–nanoscale water bridge interfaces. Each map is accompanied by its force–distance curve (average). The force–distance curves include oscillatory and monotonic terms. Additional experimental parameters: (a) *f*_2_ = 1.918 MHz, *k*_2_ = 2254 N/m, *Q*_2_ = 755, <*A*_0_> = 460 pm, <*A*_sp_> = 230 pm. (b) Left: *f*_2_ = 1.776 MHz, *k*_2_ = 1883 N/m, *Q*_2_ = 953, *A*_0_ = 453 pm, *A*_sp_ = 243 pm. Middle and right: *f*_2_ = 1.760 MHz, *k*_2_ = 2092 N/m, *Q*_2_ = 856, <*A*_0_> = 428 pm, <*A*_sp_> = 154 pm.

On mica, the 2D force maps show two hydration layers. The separation between the first and the second layer is 0.3 nm. The panels reproduce the oscillatory pattern associated with the molecular layering of water. They agree with X-ray reflectivity data^31^ and Monte Carlo simulations of water on mica.^32^

The 2D force maps show the reproducibility of the atomic-scale features. However, those maps are slightly noisier than the ones obtained when the cantilever-tip system is fully immersed in water.^5,17,25^ In addition, the force maps obtained on mica are slightly noisier than those measured on graphite. These observations are caused by different factors. First, atomic-scale resolution imaging by AFM is easier in liquid (low quality factors). Second, the presence of the adhesion well of the capillary meniscus implies the use of amplitude values larger than in bulk water (400–600 pm versus 50–200 pm). Third, the detection of hydration layers on mica by AFM methods is enhanced by using dilute electrolyte solutions (for example, 50 mM KCl).^5^ The adsorption of K^+^ from the solution stabilizes the layering of water on mica. This effect is absent in the nanomeniscus because the water comes directly from the vapor.

Our findings are summarized in Table 1. The table also includes the interlayer spacings obtained from several experiments and MD simulations on the same surfaces immersed in ultrapure water. In all the cases, the hydration layer spacings reported here agree with the measurements performed with bulk water and MD simulations. The experimental data includes different techniques such as 3D-AFM^17^ or X-ray reflectivity.^31^ We conclude that the interfacial structure of water does not depend on the volume of the confined water.

**Table 1:** Interlayer Distances Measured by 3D-AFM and Comparison with Reported Values^a^

surface	bulk water	nanomeniscus
graphite (uncontaminated)	0.3 ± 0.03 (this work)	0.3 ± 0.03
	0.32 (ref ^31^)	
	0.29 (MD, ref ^29^)	
mica	0.3 (MD, ref ^25^)	0.28 ± 0.04
	0.3 (MD, ref ^2^)	
	0.31 ± 0.04 (ref ^25^)	

a In nanometers.

We note that for graphite the data shown in the table refers to experiments performed on pristine and uncontaminated graphite surfaces. Graphite and graphite-like surfaces are prone to be contaminated by airborne hydrocarbons.^25,28^ The interaction of airborne molecules with water in the vicinity of a graphite surface leads to oscillations with larger spacings.^25^ We find it much easier to observe hydration layers inside a nanomeniscus than when the same graphite surface is fully immersed in water. In the latter case, it is more common to observe the spacings associated with hydrocarbon layers.

We apply the Kelvin equation to determine the volume of water inside the above nanoscale water bridges. Recent experimental data validate its application for menisci with diameters in the 3–4 nm range.^9,10^ The Kelvin equation establishes a relationship between the relative humidity (RH), temperature (*T*), surface tension of the liquid (γ), and the two principal curvature radii (*r*_1_, *r*_2_) (Figure 2c).
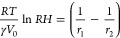
1where γ is the surface tension of the air–water interface, *V*_0_ is the molar volume of water and *R* is the gas constant. We note that the principal curvatures of the nanoscale meniscus depicted in Figure 2c have opposite signs.

Experimental and theoretical data support the geometry model of Figure 2c for the nanoscale water bridges formed by an AFM tip near a solid surface.^3,19,24^ The maximum length of the nanomeniscus is estimated from the width of the adhesion well in the FC (3–4 nm). The principal curvature radii of the meniscus are determined from eq 1. For the nanoscale water bridges formed on graphite (the contact angles θ_1_ = 0°, θ_2_ = 70°, tip radius *r*_*t*_ = 7 nm, filling angle ϕ in the 10–40° range), the diameter of nanomenisci (2*r*_2_) might vary between 5 and 9 nm. Thus, we estimate that the volume of the nanomeniscus is in the 80 to 250 nm^3^ range (*V* ≈ *hπr*_2_^2^). This volume provides a genuine nanoconfinement of water in the three spatial directions. The above volume is about 20-fold smaller than the ones reported for nanoscale water bridges.^3^

In summary, we have developed an AFM-based method to form and observe with atomic-scale resolution the interfacial structure of water under 3D nanoscale confinement. The method enables the controllable formation of very small nanoscale water bridges (250 nm^3^). The hydration layer spacing measured inside a nanomeniscus on hydrophobic (graphite) and hydrophilic (mica) surfaces are, within the experimental error, identical to those measured or on the same surfaces immersed in ultrapure water. In this respect, the confinement of water within a 3D nanoscale meniscus does not alter the out-of-plane hydration layer structure of interfacial water. Our method has the intrinsic atomic-scale resolution and nanomechanical mapping capabilities to address and eventually solve many of the scientific issues related to nanoconfined water.

## Methods

### Crystalline Surfaces

Highly oriented pyrolytic graphite (HOPG, grade ZYB) was purchased from Bruker (USA) and cleaved with adhesive tape before the experiment. Muscovite mica (Grade V-1) was purchased from SPI supplies (USA). The mica was freshly cleaved with adhesive tape before the experiments.

### Solvents

Ultrapure water was freshly obtained before the experiments (ELGA Maxima, 18.2 MΩ cm^–1^). The water’s pH value reached a value of 5.6 a few minutes after obtaining it from the machine (Hanna Instruments HI 9024).

### AFM Setup

We adapted a homemade three-dimensional AFM^25^ to study the interfacial water structures inside a nanoscale water bridge. The 3D-AFM operates in a Cypher VRS platform. Two-dimensional force maps are performed by operating the AFM in the amplitude modulation mode.^33^ The cantilever oscillates with respect to its equilibrium position by exciting it at its second resonance. At the same time, a sinusoidal signal (*f*_m_= 100 Hz) is applied to the *z*-piezo to modulate the relative *z*-distance between the sample and the tip. The *z*-piezo signal is synchronized with the *x*-displacement. For each *x*-position on the surface of the material, the tip performs a single and complete *z*-cycle. The *z*-data is read out every 20 μs and stored in 512 pixels (256 pixels half cycle). Each *x*-plane of the 2D map contains 80 points. Hence, the total time to acquire a 2D force map is 0.82 s.

The oscillation of the cantilever is driven by photothermal excitation. The free amplitude values *A*_0_ are in the range of 50–100 pm (in liquid) and 400–600 pm (in air). The feedback monitors the instantaneous amplitude and acts on the *z*-piezo to keep the lowest amplitude reached during the approach close to a fixed value (*A*_sp_ ≈ 0.75–0.35 *A*_0_). We use a feedback bandwidth of 2 kHz. It does not compensate for the small changes in the amplitude during the *z*-piezo displacement, but it is fast enough to track the surface topography.

### Force–Distance Curves

In amplitude modulation AFM the main observables are the oscillation amplitude *A* and the phase shift ϕ. Simultaneously, we record the average cantilever deflection Δ*z*. Force–distance curves (force curves) were computed^34^ from the dynamic observables *A* and ϕ (Figures 1b, 2e and f, and 3a and b) and from the average cantilever deflection Δ*z* (Figures 2a,b) as a function of the *z*-position.

The *z*-range of the reconstructed force–distance curves was slightly reduced with respect to the amplitude and phase shift–distance curves (reduced by the zero-to-peak amplitude, i.e., *A*_0_) because the force reconstruction process required an integration over the oscillation cycle.

The AFM observables are acquired by modulating the tip–surface distance *z* on different *x* positions of the surface. The amplitude modulation AFM observables (phase shifts (ϕ) and amplitudes (*A*)) have been transformed into force–distance curves by using force reconstruction methods developed for amplitude modulation AFM.^34^ To increase the signal-to-noise ratio in the force–distance curves we have calculated the value of the force by averaging the values of the observables for the different *x* positions at the same *z*. The use of AM–AFM data to reconstruct FC of solid–water interfaces is well established.^35^

Indeed, the majority of force–distance curves exhibit oscillatory and monotonically decaying terms. The community usually neglects the monotonically decaying term because the goal is to obtain the spatial frequencies of the liquid density which are given by the oscillatory term. We have recently published a contribution describing the origins of the oscillatory and monotonically decaying terms.^21^

The cantilevers (Nanoworld PPP-NCHAuD and MikroMasch 160AC-NG) were calibrated by using a contactless method included in the software of the Cypher VRS. The obtained values are summarized in the figure captions.

## Abbreviations Used

AFM: atomic force microscope
FC: force curve
HOPG: highly-oriented pyrolytic graphite
MD: molecular dynamics
RH: relative humidity
